# Examining the Mediating Role of Organisational Support on the Relationship Between Organisational Cynicism and Turnover Intention in Technology Firms in Istanbul

**DOI:** 10.3389/fpsyg.2021.606215

**Published:** 2021-05-21

**Authors:** Berat Cicek, Mehmet Ali Turkmenoglu, Mustafa Ozbilgin

**Affiliations:** ^1^Malatya Turgut Özal University, Battalgazi, Turkey; ^2^Faculty of Economics and Administrative Sciences, Muş Alparslan University, Muş, Turkey; ^3^Brunel University London, Uxbridge, United Kingdom

**Keywords:** organisational cynicism, turnover intention, perceived organisational support, structural equation modelling, social exchange theory

## Abstract

Cynicism and turnover intentions are highlighted as being detrimental to organisations’ sustainability. Drawing on the social exchange theory, this paper aims to examine the effect of organisational cynicism on turnover intention and the mediating role of organisational support on this relationship. A survey was conducted with 289 employees and managers. Data were gathered from 54 technology firms from Istanbul, Turkey, and analysed through structural equation modelling using AMOS. The findings suggest that the cognitive and affective dimensions of cynicism are significant predictors of turnover intention, and further that organisational support mediates the relationship between the cognitive and affective dimensions of cynicism and turnover intention. This research is novel in that it deepens our understanding of how detrimental workplace perceptions might affect employees’ intentions to leave their organisations and to what extent organisational support mediates this relationship in technology firms in Istanbul, Turkey. To our knowledge, no study has investigated these three variables together, as in the proposed model.

## Introduction

Cynicism, i.e., being distrustful in what is generally an unwarranted fashion, has a set of negative consequences on cynics themselves (Morf et al., 2019), their careers (Roberts and Zigarmi, 2014) and the teams and organisations (Andersson, 1996) in which they work. Similarly, turnover intention, if it remains unaddressed, leads to loss of talent and has dire consequences for the longevity and sustainability of an organisation, and emerges as a symptom of poor management practices and ineffective engagement of workers (Shuck et al., 2014). Cynicism and turnover intentions are two significant indicators of toxic workplace relations.

For a long time, researchers and practitioners have been looking for ways to understand the dynamics of cynicism and its effects on employees’ behaviours (Mignonac et al., 2018). Having reviewed the literature, turnover intention emerges as one of the most studied effects of cynicism. While cynicism and turnover intentions illustrate internal states of employees, there are personal and workplace factors that can affect the relationship between cynicism and turnover intention (Naseer et al., 2020). It is also argued that turnover is a costly process for businesses (O’Connell and Kung, 2007; Gim and Ramayah, 2020) and that certain factors, such as occupational stress, burnout and organisational cynicism, can fuel it significantly; others, e.g., job satisfaction, quality work-life and organisational support, however, effectively mitigate it. In this study, we take perceived organisational support as a mediator which is also considered as one of the workplace factors affecting employees’ turnover intention.

Several political and economic developments in Turkey are responsible for the challenging living conditions that individuals experience (Ilbars, 2000). These developments are felt in all areas of life; in other words, these hardships affect not only individuals’ private lives but also their work lives. Despite this, there is an absence of any mechanism and only partially functioning democratic processes by which individuals can express their concerns and discontent in the workplace. Challenging conditions take many forms at the workplace level for employees; for example, some suffer from burnout, while others feel alienated from their work (Oyeleye et al., 2013). In this study, we assume that the challenging conditions of employment in Turkey may cause employees to demonstrate cynical behaviour.

The high-tech industry is unique in its need and ambition to recruit and retain talented workers (Kearney et al., 2019). Cynicism and turnover threaten the longevity of firms in this sector, in which a highly skilled workforce is a critical organisational resource (Bhatnagar, 2012). From this point of view, problems can occur that need to be addressed. Accordingly, cynical attitudes amongst individuals are deemed to have effects on their turnover intention. If individuals are thinking of quitting and they are cynical at the same time, high-tech firms would struggle to recruit new staff. In this setting, we question if, and the extent to which, employees’ perceptions that there are supportive interventions in the workplace may mediate the relationship between cynicism and turnover intentions. Thus, we query if the perceived organisational support would mediate the relationship between employee cynicism and turnover intentions.

Since other researchers have investigated the relationships between cynicism and job satisfaction, ethical climate and organisational commitment, among other variables, we examine the interplay between cynicism turnover intention in technology firms in Turkey, a sector in which the demand for performance improvements is intense; the paper also discusses what organisations may do in response. As such, we examine the extent to which perceived organisational support mediates the relationship between cynicism and turnover intentions. The field study provided data from questionnaires with 289 employees and managers from 54 technology firms. The findings suggest that organisations could mobilise organisational support to mediate and counteract the negative consequences of cynicism and turnover intention. The paper starts with the definitions of key concepts and sets out the extant hypothesis. Methods and findings are presented. The paper concludes with the identification of the contribution and practical suggestions for people management.

## Organisational Cynicism

The term “cynicism” emanates from ancient Greek philosophy. Antisthenes, who was a pupil of Greek Philosopher Gorgias, defined cynicism as rejecting worldly fame, housing, desire, religion, power and dress, which have no real value in nature (Kidd, 2005). The contemporary conceptualisation of cynicism refers to a rejection of the need for social inclusion (Navia, 1999). Although there are several types of cynicism in the literature including employee cynicism, the psychological side of cynical hostility, social cynicism and cynicism toward business organisations and leaders (Andersson, 1996), we focus on organisational cynicism in this research as it is the most comprehensive form of cynicism that can be encountered at work.

Andersson and Bateman (1997) define cynicism as “both a general and specific attitude, characterised by frustration and disillusionment as well as negative feelings toward a person, group, ideology, social convention, or institution” (p. 450). It is also suggested that cynicism is a state of cynical disposition in which a cynic does not have faith in people or societal values (Kanter and Mirvis, 1989). This cynical attitude leads to the demonstration of distrust against the motives or goodness of other human beings. Pessimism, oppressive negativity and sneering are several of the main characteristics of cynical individuals, among others (Andersson and Bateman, 1997).

Organisational research highlighted the existence of cynicism in the workplace as cynicism affects organisations (Chiaburu et al., 2013; Li and Chen, 2018; Jiang et al., 2019; Aboramadan et al., 2021). Organisational cynicism has been on the rise due to mismanagement, scandal and self-centred attitudes in the workplace (Arslan and Roudaki, 2019). Therefore, there is a need to understand and examine cynicism from an organisational perspective. Dean et al. (1998), whose work is one of the most frequently cited in the associated literature, conceptualise organisational cynicism as “a negative attitude toward one’s employing organization” (p. 345). Stern et al. (1990) suggest that cynics hold a negative attitude in which they think “companies do not care about their employees and that most jobs are not worthy of a worker’s commitment” (p. 271). They further demonstrate that cynicism toward work has a negative relationship with motivation to perform better, and it is fruitless and unworthy for cynics to spend effort on the organisation.

Wanous et al. (1994) define organisational cynicism from an organisational change perspective, where they state that organisational cynicism is “a function of failed attempts at change, so that persons become pessimistic about future change and learn to blame those responsible for failing to make a change” (p. 271). Thus, the lack of agency and voice could lead to cynicism and negative attitudes toward work. Hence, it can be argued that organisational cynicism is a negative attitude of rage, pessimism, hatred, disgust, hopelessness and distrust toward the future of change in the organisation.

According to Dean et al. (1998), there are three dimensions of organisational cynicism, which include the cognitive, the affective and the behavioural dimensions. The affective dimension constitutes negative emotions of employees such as disgust, anger, distress and humiliation/shame when employees think about their organisation. The cognitive dimension refers to the belief that the organisation demonstrates a lack of honesty and integrity. The behavioural dimension points out the tendencies toward derogatory behaviour toward the organisation. We adopt this three-pronged framework for our cynicism measure.

## Perceived Organisational Support

To address their concerns, employees seek support not only from their environment but also from their organisations (Newman et al., 2012). Support from the organisation is crucial for employees to be accepted and valued as well as to be fulfilled at work (Zhai et al., 2020). Eisenberger et al. (1986) suggest that employees’ behaviours are affected by several factors, and the motivation behind employees’ efforts stems from their organisations’ actions. Perceived organisational support (POS) is described by Eisenberger et al. as “a general perception concerning the extent to which the organisation values employees’ general contributions and cares for their well-being” (Eisenberger et al., 1990: 51). It is argued that there is an exchange relationship between organisations and employees in which expectations on the part of both parties take place (Gouldner, 1960). According to the organisational support theory, employees are in an exchange relationship with their organisations for the expected rewards that they endeavoured to work for Eisenberger et al. (1986). Based on this exchange approach, organisations should support those employees who help them with their efforts (Blau, 1964; Settoon et al., 1996).

This theory also posits that employees evaluate their organisations’ policies and actions and develop a broad perception if the steps adopted by organisations are favourable to employees. For instance, when organisations’ desired goals are met, employees would expect tolerance with regard to sickness or mistakes in the future and better job conditions (Eisenberger et al., 1986). Such support from the organisation would lead to positive consequences on employees’ behaviours, e.g., increased organisational commitment, job satisfaction and reduced turnover, among others (Rhoades and Eisenberger, 2002; Bentley et al., 2016). Moreover, organisational support can be viewed as a guarantee that the appropriate support will be available to employees when they need to execute a particular duty and to handle challenges (Stefanidis and Strogilos, 2020). Therefore, it can be claimed that employees may see that their favourable actions will encourage their organisations to demonstrate support in return for their treatment (Tang et al., 2017). In our study, we consider perceived organisational support as a mediator in the relationship between cynicism and turnover intentions.

## Turnover Intention

Turnover intention is defined as an individual’s conscious desire to leave an organisation (Tett and Meyer, 1993). It is also described as thinking of quitting and looking for new employment opportunities elsewhere (Schwepker, 2001). Since turnover intention is depicted as a desire to leave the organisation for better opportunities, intention to leave is the phase immediately before actually quitting, and it is generally considered to be the intention to leave the organisation in the next 6 months (Rubenstein et al., 2019). Bluedorn (1982) suggests that those who have the intention to leave generally demonstrate actual leaving behaviour. There might be several reasons for employees to be willing to leave an organisation, which include retirement, leaving for family issues or health problems, as well as leaving to earn a higher wage and respect and/or better working conditions elsewhere (Dysvik and Kuvaas, 2010). Based on a meta-analysis, which covers studies within a period of 25 years, Fried and associates conclude that role stress has a moderate relationship with intention to leave (Fried et al., 2008). Furthermore, job dissatisfaction is the most probable consequence of employee turnover intention, or, in other words, job satisfaction has an inverse relationship with turnover intention (Zhang et al., 2019).

Employee turnover can be quite costly for organisations as it does not only endanger their strategic choices and competitiveness, but it also results in additional costs due to the process of hiring and training new personnel (Watlington et al., 2010). Organisations can reduce intention to leave by creating an ethical climate (Schwepker, 2001), an organisational learning culture (Egan et al., 2004) as well as providing fair human resources practice (Huselid, 1995). In this study, we examine turnover intention and its relationship to cynicism.

## Theoretical Framework and Hypotheses Development

The social exchange theory is one of the most suitable theories to explain employees’ intention to quit (Haar, 2006). According to this theory, individuals are constantly in the process of exchange in which the needs of each of the parties should be met by the other (Blau, 1964). The theory suggests that since individuals cannot satisfy their needs and goals alone, they must be in a mutual exchange relationship with others (Homans, 1958). Accordingly, reciprocity is the underlying norm of social exchange theory (Gouldner, 1960; Settoon et al., 1996). Blau (1964) states that the unfulfilled obligations distort the balance in a relationship of reciprocal exchange and lead to negative consequences for both parties. Based on this theory, we suggest that turnover intention is likely to occur when an organisation does not provide a reward, such as voice and autonomy, to those employees who fulfil their obligations. In parallel with breaching a prior agreement, employees would have an intention to leave the organisation (Bal et al., 2010). Similarly, recent studies found that experiencing loneliness, alienation (Öztürk Çiftçi, 2021) and exhaustion (Alola U. et al., 2019) at the workplace are positively related to turnover intention. The literature suggests that those employees who have the attributes of organisational cynicism in the workplace tend to leave their organisations (Dean et al., 1998; Leiter and Maslach, 2009). The previous research reported that organisational cynicism is a predictor of turnover intention, which is seen as a negative consequence of such (Spence Laschinger et al., 2009; Chiaburu et al., 2013). Recently, for instance, Khan (2014) conducted a study of 250 bankers in which he concluded that organisational cynicism has a direct effect on turnover intention. Therefore, based on the social exchange theory and the three dimensions of organisational cynicism, it can be proposed that:

H1: The cognitive dimension of organisational cynicism affects turnover intention positively.
H2: The affective dimension of organisational cynicism affects turnover intention positively.
H3: The behavioural dimension of organisational cynicism affects turnover intention positively.

Research illustrates that when employees think that they will get support for handling difficult jobs, they will be less likely to show cynical behaviours such as distrust and contempt, or in other words, if employees have higher cynicism toward their organisations, they perceive that they receive less organisational support from them (Treadway et al., 2004; Byrne and Hochwarter, 2008). This can be explained by the reciprocity norm of the social exchange theory, which argues that a lack of support in the exchange of employees’ effort will lead employees to show cynicism (Blau, 1964). For instance, a recent study found that lack of emotional support (e.g., incivility of supervisors) causes cynicism in the hotel industry (Alola U. V. et al., 2019). In previous studies on organisational cynicism, different variables, e.g., role stress, job burnout and workload, have been found to mediate perceived organisational support (Lynch et al., 1999; Bobbio et al., 2012; Kasalak and Bilgin Aksu, 2014). Based on those studies and the theory, we propose that:

H4: The cognitive dimension of organisational cynicism affects perceived organisational support.
H5: The affective dimension of organisational cynicism affects perceived organisational support.
H6: The behavioural dimension of organisational cynicism affects perceived organisational support.

As the social exchange theory suggests, if employees’ needs are addressed, they engage in their work in a spirit of reciprocity (Gouldner, 1960). Hence, employees are not expected to leave their organisations if they get appropriate support from them (Paillé et al., 2010; Abugre, 2017). Prior studies have shown that those employees who have higher perceived organisational support are less likely to leave their organisations (Rhoades and Eisenberger, 2002; Maertz et al., 2007). Furthermore, employees’ turnover intention has been one of the most prevalent consequences of perceived low levels of organisational support (Wayne et al., 1997; Allen et al., 2003). For instance, recent research conducted by Teoh et al. (2016) investigated the impact of unsupportive and supportive manager behaviours on 252 United Kingdom-based company employees. They concluded that supportive manager behaviours reduce employees’ intention to quit. Thus, we hypothesise that:

H7: Perceived organisational support affects turnover intention negatively.

Several studies (Khan, 2014; Nazir et al., 2016) have shown that lower job performance, job dissatisfaction, lesser organisational commitment and higher intention to leave the organisation are negative consequences of employee cynicism. In a similar vein, several studies have postulated a negative effect between employees’ cynicism and their intention to quit (Leiter and Maslach, 2009; Volpe et al., 2014). At the same time, data from several sources have identified that a decreased level of intention to quit is associated with higher perceived organisational support (Eder and Eisenberger, 2008; Dawley et al., 2010). Thus, we hypothesise that:

H8: POS mediates the relationship between the cognitive dimension of organisational cynicism and turnover intention.
H9: POS mediates the relationship between the affective dimension of organisational cynicism and turnover intention.
H10: POS mediates the relationship between the behavioural dimension of organisational cynicism and turnover intention.

**FIGURE 1:**
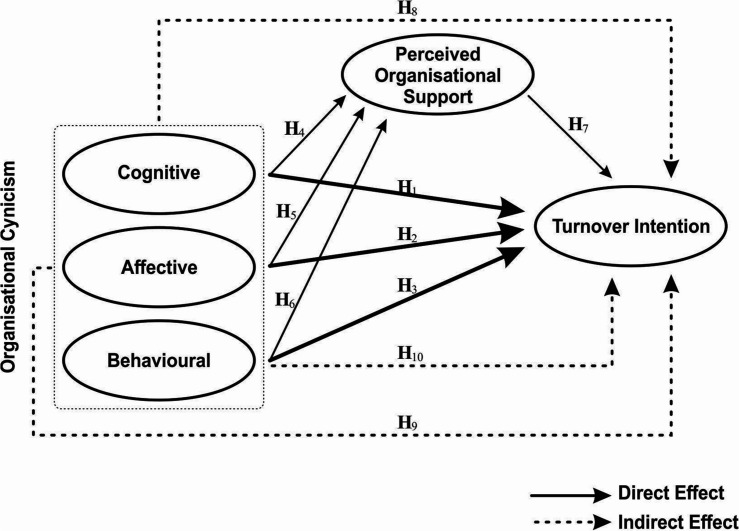
Proposed model of organisational cynicism, perceived organisational support and turnover intention.

Our proposed model and hypotheses with direct and indirect effects are illustrated in Figure 1.

## Methodology

In this research, we adopted a quantitative approach to test our hypotheses. We gathered data from technology companies in order to examine the aforementioned relationships between cynicism, perceived organisational support and turnover intentions. We conducted a survey study with employees and managers of 54 small- and medium-sized technology firms operating in the city of Istanbul in 2019. We have chosen Istanbul as the site of the study as it is the largest and the most diverse city of Turkey, with individuals who come from different cultures and from all regions of Turkey. We chose maximum variation sampling among purposive sampling techniques, as this sampling was appropriate to serve the aim of the study. To elaborate it, we considered that the participants from different age, gender, position, tenure and work setting would better fulfil the purpose of the research. Having diverse characteristics of participants from 54 different technology firms in Istanbul provided different views on the constructs mentioned above.

We contacted the CEOs or managers of the technology firms by ringing them or sending emails to them for research access. Those who accepted to cooperate allowed us research access to their firms. We handed out questionnaires to participants after getting permission from CEOs or managers and consent from individual participants. Each questionnaire contained a cover page explaining the purpose and description of the study and our assurances of anonymity and confidentiality. The cover page also included the names and contact details of the researchers. We indicated that the data would be gathered in an anonymous manner, shared only by the research team; no raw data would be shared with management. Participants were also informed that they could leave any question unanswered in order to ensure free and safe participation. We made sure that participants were kept confidential and used purely for academic purposes. Moreover, we assured the respondents that participation in the study was voluntary, and there were no right or wrong answers.

Within this context, we examined the effects of cynical behaviours of technology firms’ employees and managers on their turnover intentions and the mediating effect of perceived organisational support on this relationship. We used structural equation modelling (SEM) to test the multiple causal relationships. According to Iacobucci et al. (2007), SEM is a sophisticated tool that can be used to statistically examine the causal relationships of theoretical and empirical studies. In particular, the SEM approach is more useful than the regression approach when investigating the mediating roles of constructs. For this reason, SEM was preferred as the method of analysis for our research.

### Participants

We contacted 135 technology firms in Istanbul via email and telephone to ask if they would like to participate in the study. Before they give permission, CEOs and/or managers often requested a copy of the questionnaire and further particulars of the study. We provided the aim of the study and ethical assurances, along with a copy of the questionnaire. Fifty-four of the organisations that we approached granted permission to distribute the questionnaire. In order to increase the response rate, the drop-off/pick-up method was chosen (Lovelock et al., 1976). We also hired a postgraduate student to help us distribute and collect the questionnaires. We distributed 419 questionnaires and received 310 completed questionnaires from employees and managers, achieving a response rate of 73.98%. However, 11 out of the 310 questionnaires were not included in the final evaluation as either most items were left blank or all items were answered with the same pattern. Also, 10 questionnaires were not taken into consideration as they constituted outliers in Cook, Leverage, and Mahalanobis’ distance and boxplot determination. Thus, the data collected from 289 employees and managers were considered suitable for statistical analysis. Accordingly, the sampling consisted of n = 158 women (54.7%), n = 131 men (45.3%), n = 97 managers (33.6%) and n = 192 employees (66.4%). The mean age of the participants was 32.69 years, and the mean of their organisational tenures was 4.06 years.

### Measurement Tools

Organisational Cynicism Scale: To measure organisational cynicism as the independent variable of the study, we used the scale developed by Brandes et al. (1999) as adapted to Turkish by Kalağan (2009). The scale consists of three dimensions (cognitive, affective and behavioural) and 13 items. There are five items in the cognitive dimension and four items in affective and behavioural dimensions. Sample items: “I get angry as long as I think of the institution at which I work,” “I complain about the goings-on at work to my friends outside the institution at which I work.” The Cronbach alpha coefficients were 0.881 for the cognitive dimension, 0.936 for the affective, and 0.705 for the behavioural.

Perceived Organisational Support Scale: To measure the perception of organisational support, we used the “perceived organisational support” scale as the mediating variable of the research, as developed by Eisenberger et al. (1986) and adapted by Armstrong-Stassen and Ursel (2009) and that included 10 items. Since this scale was in English, two professional bilingual translators translated the scale from English to Turkish using Brislin’s (1980) parallel blind technique. Armstrong-Stassen and Ursel (2009) reverse-coded the items numbered 2, 6, and 9 in their study. While creating a questionnaire form in our study, we followed this rule. Sample items: “The organisation strongly considers my goals and values,” “The organisation feels there is little to be gained by employing me for the rest of my career (reverse scored).” The Cronbach alpha coefficient was 0.954 for the scale.

Turnover Intention Scale: We measured this variable using the three-item scale of turnover intention developed by Hom et al. (1984) and adjusted by Mitchell et al. (2001). The items of this scale were in English and so were translated into Turkish using the same blind technique as described above (Brislin, 1980). One sample item is, “Do you intend to leave the organisation in the next 12 months?” The Cronbach alpha coefficient was 0.852 for the scale.

All scales were represented using a five-point Likert scale (1 = strongly disagree, 5 = strongly agree). Before distributing the questionnaires to the participants, we conducted a pilot survey with 25 employees and 25 managers. After being convinced that the questionnaires did not have any errors, we decided to proceed to collect data. Statistics of the mean, standard deviation and correlation of the scales are given in Table 1.

**TABLE 1:** Descriptive statistics.

	Mean	SD	1	2	3	4	5
1. Cognitive	2.78	1.00	**0.812**				
2. Affective	3.03	1.15	0.650***	**0.888**			
3. Behavioural	2.80	1.07	0.533***	0.657***	**0.781**		
4. Perceived organisational support	3.16	1.07	−0.503***	−0.437***	−0.265***	**0.851**	
5. Turnover intention	2.59	1.07	0.577***	0.595***	0.328***	−0.565***	**0.815**

***Correlation is significant at the 0.001 level (2-tailed). The square roots of the AVEs are highlighted in bold.

### Common Method Bias Test

Since this study is cross-sectional in nature, the common-method variance (CMV) would emerge as an issue when analysing the data. CMV, which causes measurement errors, refers to the variance that is attributable to the shared measurement method rather than to the constructs (Podsakoff et al., 2003). To test for CMV, we used the single factor test described by Harman (1967). Accordingly, we examined all the items under one factor without using the rotation method. The total variance explained by the created factor was 34.74%. Since this was below 50%, we observed that there was no common method error (Kline, 2005).

After the test, we used the common latent factor (CLF), which comprises adding a new latent common variable factor in connection with all observed elements. If the inclusion of a new common latent factor does not enhance the fit indices to any considerable extent, one can argue that common method variance is not a problem. After including the CLF latent variable in our model, we observed that there were no changes in fit indices, which showed that the common method error does not exist in our method (Eichhorn, 2014).

## Findings

### Measurement Model

We analysed the data using the AMOS 24 software in the “maximum likelihood estimation” mode using the two-step structural equation modelling approach, as suggested by Anderson and Gerbing (1988). Initially, the data screening process was carried out using the SPSS v25 software. We assigned a series mean for missing values in the responses. Later, we observed quite normal distributions in terms of skewness for our latent factor indicators as well as for other variables (for example, age, experience). The skewnesses for all variables were well below ± 1. Furthermore, we observed kurtosis for the fifth item of cognitive dimension belonging to organisational cynicism. In factor analysis, as the factor value of this item was below 0.50, we excluded it from the measurement modelling. For this reason, we stated that the normality was within the acceptable range for all the variables tested in the structural model (Field, 2009).

To control the factor composition of variables, exploratory factor analysis (EFA) was performed using the Promax rotation method. The Kaiser–Meyer–Olkin (KMO) value was found as 0.916 at the executed EFA, and the Bartlett test was significant (χ^2^ = 5523.991; *df* = 325; *p* < 0.001). When the five-dimensional composition of the pattern matrix was examined, we identified that the factor loadings of items (which were codified as Cognitive5, Behaviour1, Behaviour2, POS3 and POS9) were below the 0.50 threshold. Thus, these items were excluded from the analyses. Afterward, multicollinearity was checked. It was observed that the variance inflation factors (VIFs) for all variables were below 3. These values showed that our results are quite reasonable as VIF values are accepted up to 5 (Craney and Surles, 2002).

Fornell and Larcker (1981) contend that prior to testing, the measurement model must have satisfactory discriminant and convergent validity and reliability to ensure a significant relationship in the measurement model. As a result of confirmatory factor analysis (CFA), the model’s goodness-of-fit indices were observed to be in an acceptable range (Hu and Bentler, 1999). χ^2^ = 396.921 (179); χ^2^/*df* = 2.217; RMSEA = 0.065; GFI = 0.913; AGFI = 0.918; CFI = 0.956. Subsequently, we tested whether the scales used in the measurement model were providing convergent and discriminant validity. According to Fornell and Larcker (1981), all factor loadings should exceed 0.50 considerably for the scales to have convergent validity. The average variance extracted (AVE) should exceed unexplained variance (AVE > 0.50) (Bagozzi and Yi, 1988), and the factor composite reliability (CR) should be greater than or equal to 0.60 (Fornell and Larcker, 1981). The scales used for the convergent validity tests are given in Table 2. It was found that the scales in the measurement model provided a quite strong convergent validity. To provide discriminant validity, estimated values for the extracted variances (variance-extracted estimates) should exceed the estimated values of the squared correlation (Fornell and Larcker, 1981). It can be seen from Tables 1, 2 that the AVEs for all variables were greater than the squared correlations. Moreover, the AVEs were greater than the maximum-shared variances (MSVs), and this supports the idea that discriminant validity for all variables was achieved according to the Fornell and Larcker criterion. However, as all α values and CRs of the variables were greater than 0.70, the validity of scales was also achieved.

**TABLE 2:** Results of the CFA.

Items	Factor loading	α	CR	AVE	MSV
**Cognitive**		**0.881**	**0.884**	**0.659**	**0.423**
Cognitive1	0.649				
Cognitive2	0.809				
Cognitive3	0.886				
Cognitive4	0.880				
**Affective**		**0.936**	**0.937**	**0.788**	**0.431**
Affect1	0.860				
Affect2	0.884				
Affect3	0.889				
Affect4	0.916				
**Behavioural**		**0.705**	**0.748**	**0.610**	**0.431**
Behaviour3	0.579				
Behaviour4	0.941				
**Perceived organisational support**		**0.954**	**0.954**	**0.725**	**0.320**
POS1	0.854				
POS2	0.684				
POS4	0.865				
POS5	0.894				
POS6	0.855				
POS7	0.908				
POS8	0.887				
POS10	0.842				
**Turnover intention**		**0.852**	**0.855**	**0.664**	**0.354**
ItL1	0.777				
ItL2	0.823				
ItL3	0.843				

*CR, composite reliability; AVE, average variance extracted; MSV, maximum-shared variance. All loadings are significant at the 0.001 level. The bold values represent the alpha, CR, AVE and MSV values of the variables.*

### Structural Model

We established structural equation modelling through AMOS 24 (maximum likelihood estimation) to test our hypotheses, in which we tested both direct and indirect relationships. To test the mediation effect, we adopted Baron and Kenny’s (1986) approach, which is widely used to test the indirect relationships in quantitative studies. It is argued that this approach yields good results; however, it does not provide p-values for indirect effects (MacKinnon et al., 2002). For this reason, Mallinckrodt et al. (2006) propose that calculating confidence intervals for population parameters in bootstrap analyses provides accurate results. In this context, the steps suggested by Baron and Kenny (1986) were followed for the bootstrapping method during analysis of the mediation effect. We adjusted the bootstrapping sample to be 5,000 for our study.

**FIGURE 2:**
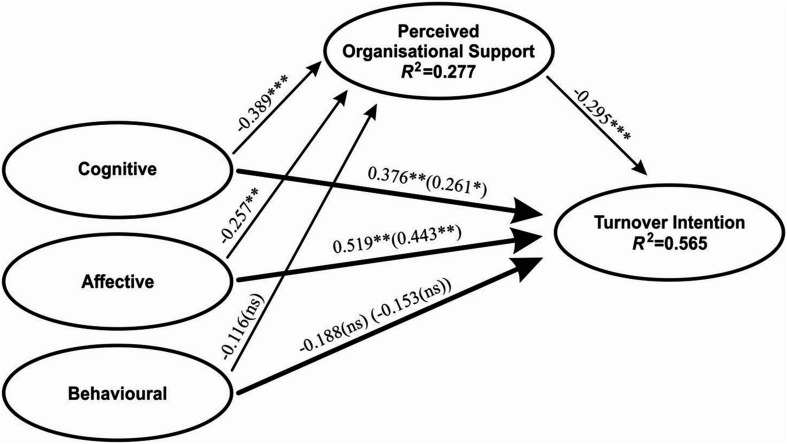
Proposed research model. ***p < 0.001, **p < 0.01, *p < 0.05; ns, not significant, parenthetical = after adding mediating variable coefficients.

We ran a model that corresponds to our proposed model to assess the hypotheses related to direct and indirect effects. The operated model showed acceptable goodness-of-fit indices χ^2^ = 323.959 (173); χ^2^/*df* = 1.873; RMSEA = 0.055; GFI = 0.911; AGFI = 0.881; CFI = 0.970. Direct effect assessments of the model are given in Table 3, and indirect effect assessments are given in Table 4 (the results are also illustrated in Figure 2). Accordingly, among the subdimensions of organisational cynicism, cognitive (β = 0.261; *p* < 0.01) and affective (β = 0.443; 0.001) had positive and significant effects on turnover intention. On the basis of these results, hypotheses H1 and H2 were accepted. It emerged that the behavioural (β = −0.153; *p* > 0.05) dimension had no significant effect on turnover intention, according to which hypothesis H3 was rejected.

**TABLE 3:** Direct effects.

Hypotheses	Coefficient	SE
H_1_: Cognitive → TI	0.261**	0.082
H_2_: Affective → TI	0.443***	0.090
H_3_: Behavioural → TI	−0.153	0.080
H_4_: Cognitive → POS	−0.389***	0.084
H_5_: Affective → POS	−0.257**	0.093
H_6_: Behavioural → POS	0.116	0.082
H_7_: POS → TI	−0.295***	0.062

*p < 0.05; **p < 0.01; ***p < 0.001.

**TABLE 4:** Mediation analysis.

Hypotheses	Total effect β	Direct effect β	Indirect effect β	Mediational situation
H_8_: Cognitive → POS → TI	0.376**	0.261*	0.115***	Partial mediation
H_9_: Affective → POS → TI	0.519**	0.443**	0.076*	Partial mediation
H_10_: Behavioural → POS → TI	−0.188 (ns)	−0.153 (ns)	−0.034 (ns)	No mediation

ns, not significant. *p < 0.05; **p < 0.01; ***p < 0.001.

We observed that the cognitive (β = −0.389; *p* < 0.001) and affective (β = −0.257; *p* < 0.01) dimensions had negative and significant effects on perceived organisational support. Based on this result, hypotheses H4 and H5 were accepted. It was revealed that the behavioural (β = 0.116; *p* > 0.05) dimension had no significant effects on perceived organisational support. Thus, hypothesis H6 was rejected. We observed that perceived organisational support (β = −0.295; *p* < 0.001) had a negative and significant effect on turnover intention. Accordingly, hypothesis H7 was supported.

Hypothesis H8 assumes that perceived organisational support mediates the relationship between the cognitive dimension and turnover intention. According to the bootstrapping analysis, we witnessed that perceived organisational support had a partial mediating effect on this relationship. Since it had a significant direct effect, hypothesis H8 was supported. Hypothesis H9 proposes that perceived organisational support has a mediating effect on the relationship between the affective dimension and turnover intention. As a consequence of our analysis, we found that perceived organisational support has a significant indirect effect in this relationship, and that there was an associated partial mediation. Accordingly, hypothesis H9 was accepted. Hypothesis H10 assumed that perceived organisational support has a mediating effect on the relationship between the behavioural dimension and turnover intention. However, since the direct and indirect effects of the behavioural dimension were insignificant, hypothesis H10 was rejected.

## Discussion

In the wake of the COVID-19 pandemic, there is a growing recognition that it is imperative for organisations and individuals to manage negative organisational phenomena such as cynicism, stress, redundancies and burnout in order to survive the dire consequences of the pandemic (Abdelhafiz et al., 2020; Charoensukmongkol and Phungsoonthorn, 2020; Yıldırım and Solmaz, 2020). One of the ways that organisations manage such crises has been to offer interventions and support mechanisms in order to retain key staff, while individuals often develop alternative means of resilience and coping mechanisms to tackle the negative consequences of crises. Yet, the traditional ways in which organisations and individuals have coped with crisis situations that generate negative organisational affect and consequences in the past often involved psychological interventions which require face-to-face contact; this is currently, of course, extremely difficult due to widespread use of lockdown and curfew measures. We predict that organisations may need to find alternative means to provide support for their staff beyond the traditional through the creative use of virtual interfaces. Although our study was conducted prior to the COVID-19 pandemic, the way in which the pandemic has engendered negative organisational outcomes highlights the relevance of our key finding, that perceived organisational support could be used as a means to lessen the impact of one such negative organisational phenomenon, namely, cynicism, on turnover intentions. Heeding the resurgence of cynicism in organisations in the context of the COVID-19 pandemic, we identify that the perceived organisational support, which could be attained in myriad of ways including in-person and virtual interventions, could lessen individuals’ turnover intentions.

In other words, drawing on the social exchange theory (Blau, 1964), this study aimed to investigate the influence of organisational cynicism on turnover intention and the mediating role of organisational support. The results demonstrated that cognitive and affective dimensions of cynicism have positive and significant effects on turnover intention, yet the behavioural dimension is apparently insignificant. The cognitive and affective dimensions affected perceived organisational support in a negative manner, while the behavioural dimension did not have any apparent significant effect. Moreover, perceived organisational support adversely affected turnover intention. We demonstrated that organisational cynicism causes turnover intention, and perceived organisational support can mediate turnover intention in organisations, as supported by several studies (Allen et al., 2003; Loi et al., 2006).

The indirect effect analysis of our study shows that perceived organisational support mediates the possible negative impact of cognitive and affective cynicism on turnover intentions. However, the same mediation effect is not evident in the relationship between cynical behaviours and turnover intentions. As Byrne and Hochwarter (2008) demonstrate, perceived organisational support only mediates cynicism at work. Our study suggests that mediating cynical behaviours requires more than perceived support to change behaviours at work. Behaviours are harder to change than cognition and affect. Our study concurs with the findings of Kasalak and Bilgin Aksu (2014) in that a lack of perceived organisational support can also lead to more cynical behaviours at work. One possible interpretation of this finding could be that cynical behaviours require more substantial organisational interventions than support mechanisms alone.

## Theoretical Contributions and Implications

Our research makes a primary contribution to the literature. For instance, Cartwright and Holmes (2006) suggest that changing the meaning of work and engaging with emotions and cognition at work could help move workers out of cynical frames of thought and affect. Since organisational cynicism increases the turnover intention of employees, we contribute to Cartwright and Holmes’ (2006) work by showing how detrimental workplace perceptions might affect employees’ intentions to leave their organisations and to what extent organisational support mediates this relationship in technology firms. Our study fills a gap in the literature by integrating the organisational cynicism model of Brandes et al. (1999), the organisational support frame of Armstrong-Stassen and Ursel (2009) and the turnover intention conceptual model of Mitchell et al. (2001). We found that perceived organisational support mediates the impact of negative affective and cognitive cynicism on employees’ intention to leave their firm. Yet, the results do not confirm that perceived organisational support is a significant mediator of the relationship between cynical behaviours and turnover intentions.

The technology sector is affected by employees’ behaviour due to its very nature, e.g., the pessimistic attitudes of employees in their workplaces affect their creativity. Since the technology sector is a sector where creativity is highly prized, cynical behaviours are extremely undesirable (Dockel et al., 2006). Cynicism and turnover intention include feelings such as distress, frustration and hopelessness (Stern et al., 1990). Therefore, cynicism and turnover intention may negate creativity in organisations. One reason for these feelings might be the lack of trust employees have in the organisation (organisation strategies and policies), colleagues, stakeholders and managers. From this point, the findings of this study may guide technology firms’ managers and stakeholders to consider cynicism and turnover intention as part of their talent acquisition and retention strategies. In the context of Turkish technology firms, managers and stakeholders may foster environments and climates of trust in their workplaces. This perception of trust would give employees the feeling and the awareness that they are supported by their organisations. Perceived support would help employees to stay in their organisations.

As can be seen from the results of this study, perceived organisational support can prevent cynical behaviours before actual turnover intention arises. Besides, we recommend that managers ensure that employees should participate in decision-making processes about themselves. Managers should treat employees fairly and equitably and provide them with a climate in which they can express their feelings and views.

## Limitations and Future Directions

Despite the stated contribution to the literature on cynicism, turnover intention and organisational support, this study is not without its limitations. Our study illustrates the relationship between cynicism, perceived organisational support and turnover intention. As there is a significant drive toward reducing turnover intention in the high technology sector, and cynicism has been identified as a major cause of turnover intention, our study shows that perceived organisational support could be the way to tackle the effect of cynical emotions and cognition on turnover intention. Despite this, there may be other interventions through which an organisation could address cynicism and turnover intention. As such, there is a need for further research to identify the root causes of cynicism to examine how behavioural forms of cynicism could be tackled in organisations. It is clear that perceived organisational support is not a sufficient mechanism for this. Thus, we suggest an investigation of different variables such as authentic and servant leadership styles, job satisfaction, supervisor support, peer support, high-quality leader–member exchange relationships, human resource management practises and self-regulation amongst employees as mediators in the relationship between cynicism and turnover intention for future research.

In addition to this, although we adopted a cynicism scale that has been frequently used in the Turkish context, some items had to be removed from the scale due to problems relating to kurtosis and factor loadings. This may be viewed as a limitation. In particular, the lack of significance we found in the dimension of behaviour may be attributed to this limitation. This may also be because participants were not entirely honest about, or possibly even aware of, an impact on their behaviour.

## Conclusion

As mentioned at the outset, the turnover intention has been a crucial problem for firms to tackle. In particular, it is quite challenging to keep employees within high technology firms, as talented individuals might easily find better opportunities. It is argued that one antecedent of employee turnover intention is organisational cynicism. Although cynical employees are likely to have higher turnover intentions, we found that if employees feel that there is organisational support, they are less likely to gain the intention to leave their organisations. Thus, organisations should provide extra support for their employees if they wish to reduce turnover intentions.

## Data Availability Statement

The raw data supporting the conclusions of this article will be made available by the authors, without undue reservation.

## Ethics Statement

The studies involving human participants were reviewed and approved by Scientific Research and Publication Ethics Board of Mus Alparslan University. The patients/participants provided their written informed consent to participate in this study.

## Author Contributions

All authors listed have made a substantial, direct and intellectual contribution to the work, and approved it for publication.

## Conflict of Interest

The authors declare that the research was conducted in the absence of any commercial or financial relationships that could be construed as a potential conflict of interest.
